# A Prosthetic Solution to Poorly Placed Implants in the Posterior Mandible

**DOI:** 10.1155/2018/1327230

**Published:** 2018-11-25

**Authors:** Michele Albino de Oliveira Treml, Laerte Balduíno Schenkel, Guenther Schuldt Filho

**Affiliations:** ^1^São Leopoldo Mandic University, Campinas, Brazil; ^2^Brazilian Society of Oral Rehabilitation, Belo Horizonte, Brazil; ^3^Santa Catarina South University, Palhoça, Brazil

## Abstract

The objective of the present study is to find a solution for patients who have multiple implants that are poorly placed in the posterior mandible and require a solution to be rehabilitated, taking into account some diagnostic principles such as maintenance of the occlusal plane, maintenance of correct dental arch inclination, and adequate vertical dimension.

## 1. Introduction

The rehabilitation of edentulous patients has always been a great challenge for dentistry, and it was then that implantology emerged as a viable alternative for the replacement of dental absences. Through Brånemark and his team in 1969, it was possible to conceptualize osseointegration as a direct structural and functional connection between the organized vital bone and the surface of a titanium implant capable of receiving functional load [1].

Since the 1980s, osseointegrated implants have begun to have a more routine presence in dentistry. In a more secure and predictable way, the planning of implant prostheses began to be performed by two forms of retention: screwed or cemented. In order to choose the form of retention, the rehabilitator must take into account the characteristics, indications, contraindications, and advantages and disadvantages of each of these prosthetic options in order to solve the clinical cases with prostheses on implants with excellence [2].

Several positive and negative aspects revolve around cementation or screw fitting of the implant-prosthesis connection [3]. The greatest advantage of screw prostheses is undoubtedly the reversibility that they offer [4]. In addition, procedures such as hygiene, repair, and maintenance are eventually necessary and become easier to perform in cases of a screwed prosthesis [5]. Problems such as angulation of the inadequate implant are more easily solved with cemented prostheses, in addition to situations such as limited mouth opening, implant placed in the distal position or located in the posterior region, and aesthetic factor which contraindicates the screwed prosthesis and require a cemented prosthesis [6].

Cases of difficult resolution, mainly related to posterior implants with inadequate angulation, make us reflect on the most appropriate way to restore. Several clinical studies on screwed and cemented techniques are described in the literature [7–11], but few with a combined technique that unites the main advantages of each system are available.

The present article reports a clinical case with multiple implants installed in the posterior region of the mandible with inadequate angulation and that required a different planning at the time of rehabilitation in relation to the retention system to meet the functional and aesthetic needs of the patient. It will be reported in the present article how we designed a combined prosthesis: screw retained and cement retained in order to optimize the advantages of both implant-supported prosthesis retention techniques and resolve cases of implants that are poorly positioned in the posterior region of the mandible.

## 2. Case Report

The patient, 47 years old, female, systemically healthy, and nonsmoker, presented in the Laerte Schenkel Residency Course of Dentistry, a private clinic, where the case was carried out, requiring the rehabilitation of MultiPlus implants already installed in the posterior region of the mandible corresponding to the dental elements 35, 36, 37, 46, and 47. The main complaint focused on masticatory difficulty in the already-existing crowns, frequent mobility of these crowns, absence of aesthetics, and difficulty in sanitizing. At the time of the anamnesis, screwed metal-ceramic crowns joined in the implant region, mucositis, food retention, and degradable odor were found.

**Figure 1:**
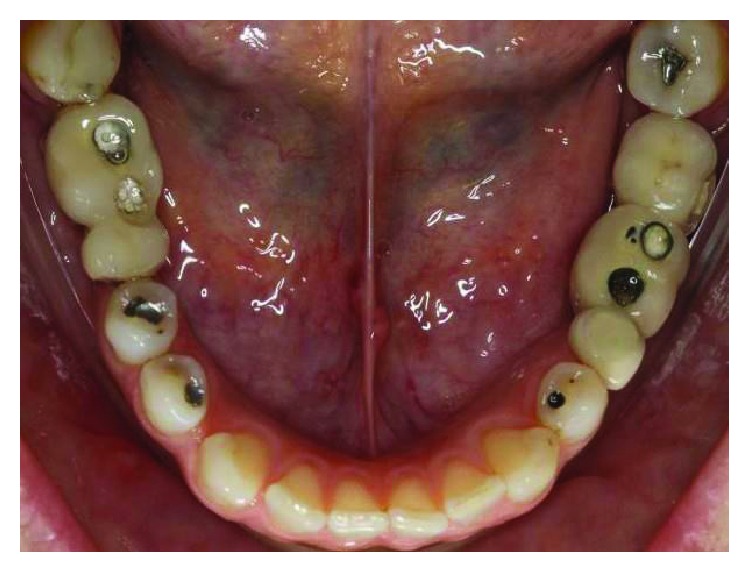
The initial clinical case, occlusal view. Source: DOCEO-SC.

**Figure 2:**
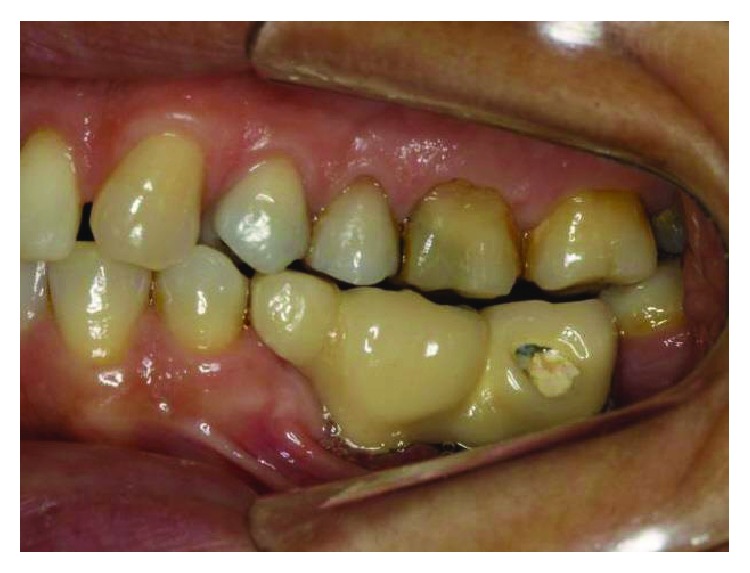
The initial clinical case, lateral view, posterior left region. Source: DOCEO-SC.

**Figure 3:**
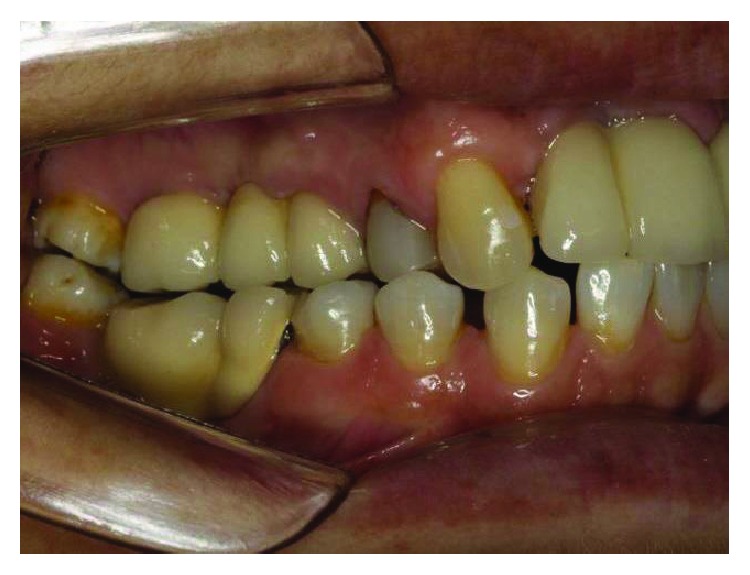
The initial clinical case, lateral view, posterior right region. Source: DOCEO-SC.

**Figure 4:**
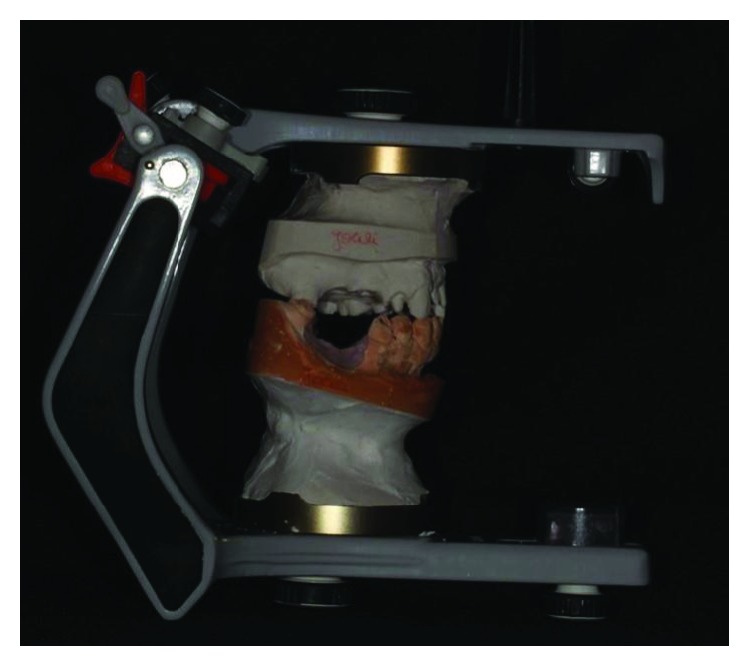
Articulator mounting. Source: own authorship.

**Figure 5:**
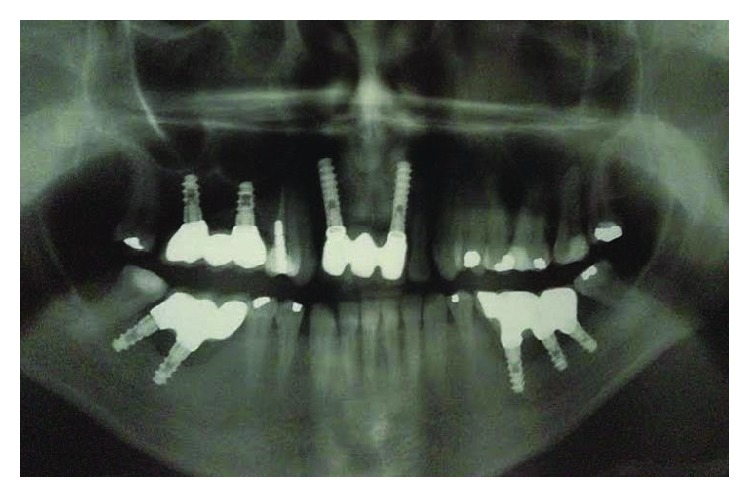
Panoramic radiography. Source: DOCEO-SC.

Before the planning of the new prosthetic rehabilitation, a working protocol was established with diagnostic tools: initial photographic documentation (Figures 1–3) of the patient, work models mounted on a semiadjustable articulator (Figure 4), and evaluation of panoramic radiography (Figure 5). With the articulated work models, the unevenness of the occlusal plane was evaluated (Monson's plaque-Monson's theory [12]); alteration of vertical dimension and crowns outside the dental arch were found. In the radiographic evaluation, the inadequate angulation of the implants and the connection of the installed implants were verified: external hexagons which were screwed directly on the implant.

**Figure 6:**
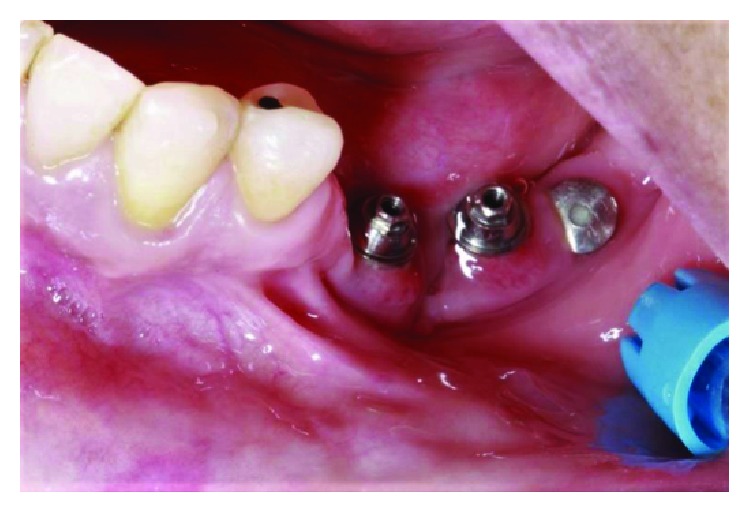
Minipillar installation in the left posterior region with burying of most posterior implant. Source: own authorship.

**Figure 7:**
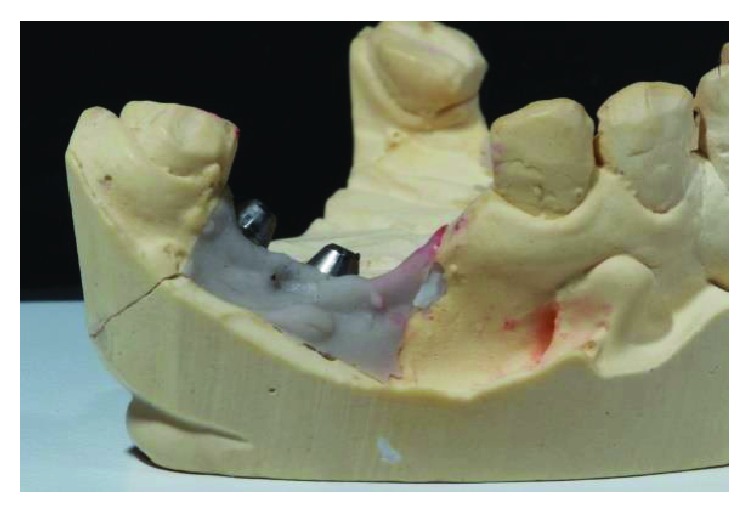
The mold obtained from regions 46 and 47. Source: own authorship.

**Figure 8:**
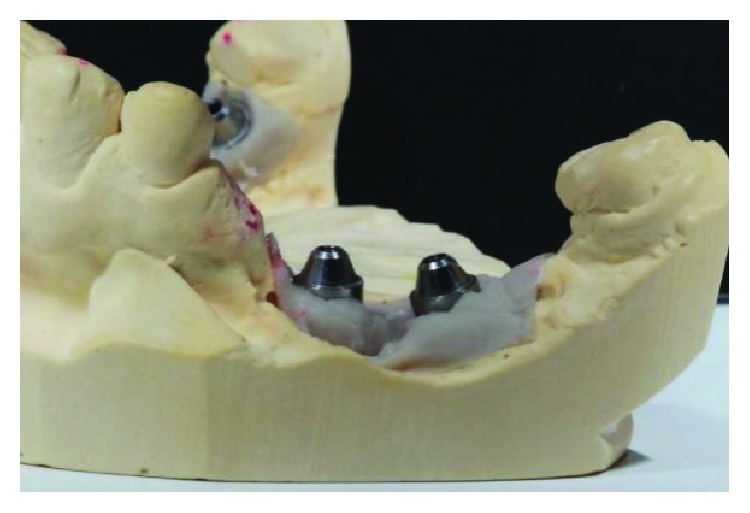
The mold obtained from regions 35 and 36. Source: own authorship.

In the first stage of the treatment, the installation of minipillars (Neodent brand) on the implants (posterior region right and posterior region left) was chosen, with the proposal to remove the prosthetic connection from intimate contact with the gingiva, thus providing a better hygiene condition and improvement with the mucositis. In the posterior region of the mandible, a lack of keratinized gingiva is common so the installation of minipillars favors gingival health in this region. It was not possible to maintain the most posterior implant of the left side in the planning of the new crowns since it was very vestibularized and outside the patient's dental arch, jeopardizing the hygiene of the new crowns so it was decided to bury it (Figure 6). The model obtained from regions 46 and 47 can be seen in Figure 7 and the model obtained from regions 35 and 36 can be seen in Figure 8.

**Figure 9:**
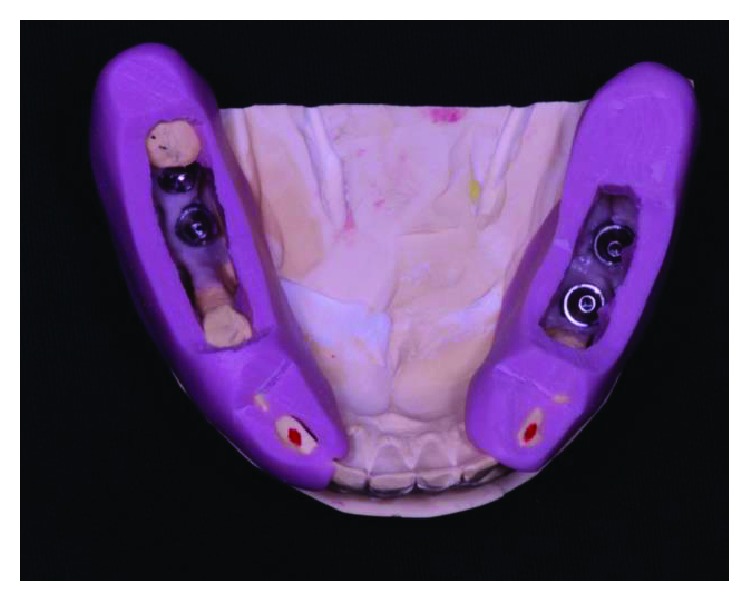
Conference of positioning of implants in the arch. Source: Dental Design Laboratory.

**Figure 10:**
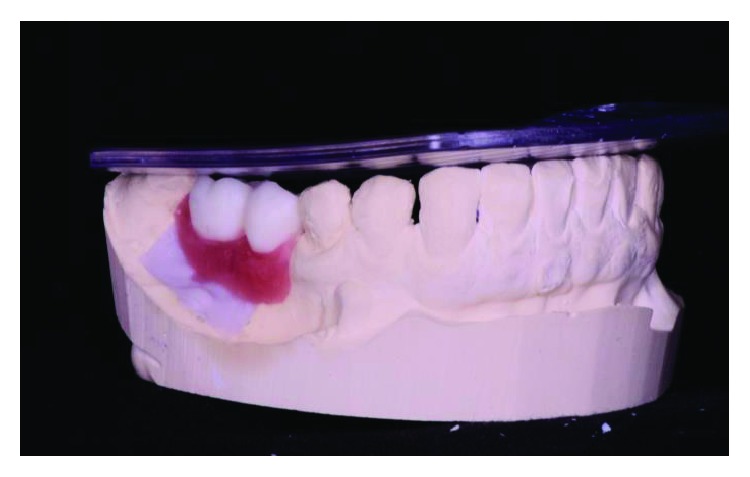
Waxing of the right posterior region according to the occlusal plane (Monson's plaque). Source: Dental Design Laboratory.

**Figure 11:**
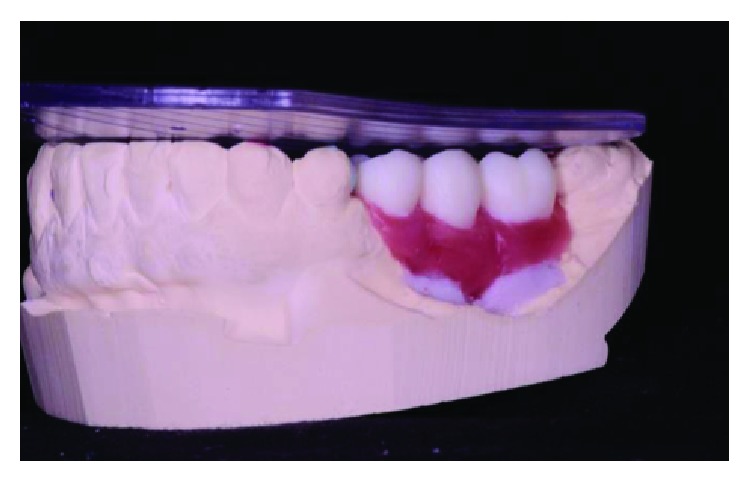
Waxing of the left posterior region according to the occlusal plane (Monson's plaque). Source: Dental Design Laboratory.

**Figure 12:**
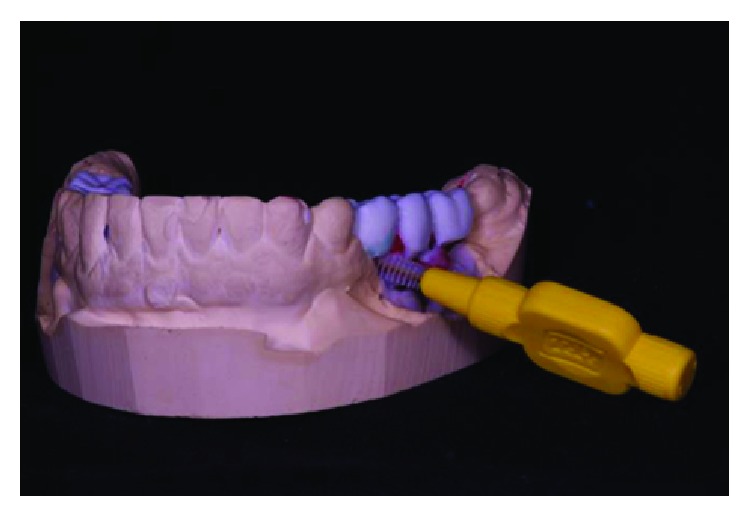
Predicting hygiene. Source: Dental Design Laboratory.

The laboratory phase was performed by a dental technician, and each step was rigorously followed, from the conference of the moldings and models obtained (Figure 9), diagnostic waxing (Figures 10 and 11), maintenance of the occlusal plane, and sanitation planning of the crowns (Figure 12).

**Figure 13:**
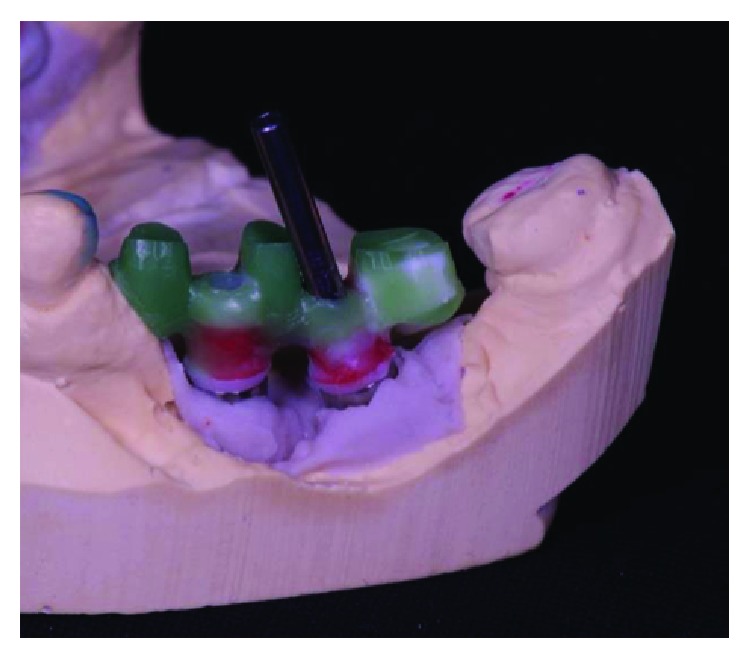
Metal structure waxing predicting the insertion of the key. Source: Dental Design Laboratory.

**Figure 14:**
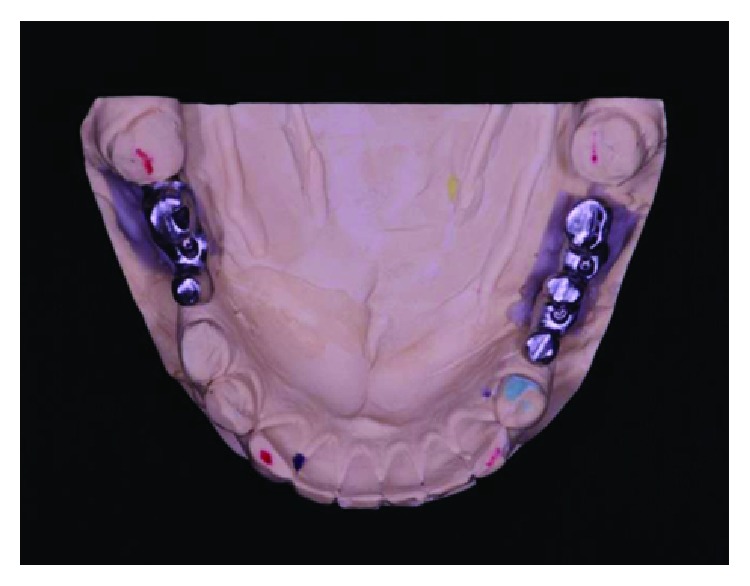
Alloy framework (cobalt-chromium) casted according to the primary structure waxing. Source: Dental Design Laboratory.

**Figure 15:**
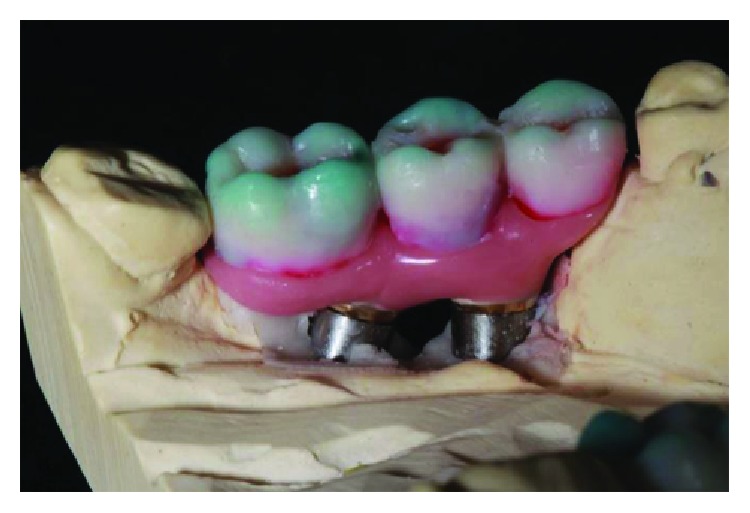
New waxing of the crowns and gingiva. Source: Dental Design Laboratory.

**Figure 16:**
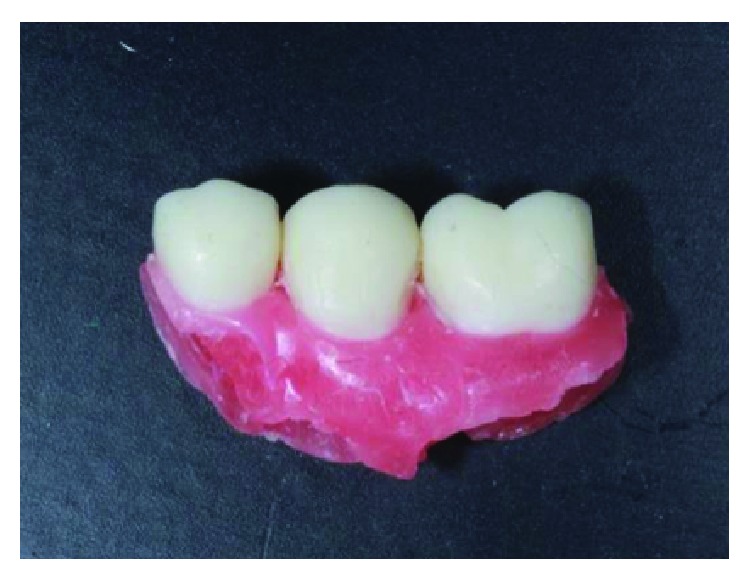
New waxing of the crowns and gingiva. Source: Dental Design Laboratory.

The authors' project for a new prosthesis treatment plan consisted of joining in one study the main advantages of cemented prosthesis: aesthetics and passivity and the main advantage of screw prosthesis: reversibility. The proposal to perform cemented-retained and screw-retained crowns in multiple prostheses began by making a waxing of the future alloy primary structure framework (Figure 13), predicting the insertion of the key. Then the alloy framework (cobalt-chromium) was casted according to the waxing (Figure 14). New waxing of the crowns and gingiva was performed (Figures 15 and 16).

**Figure 17:**
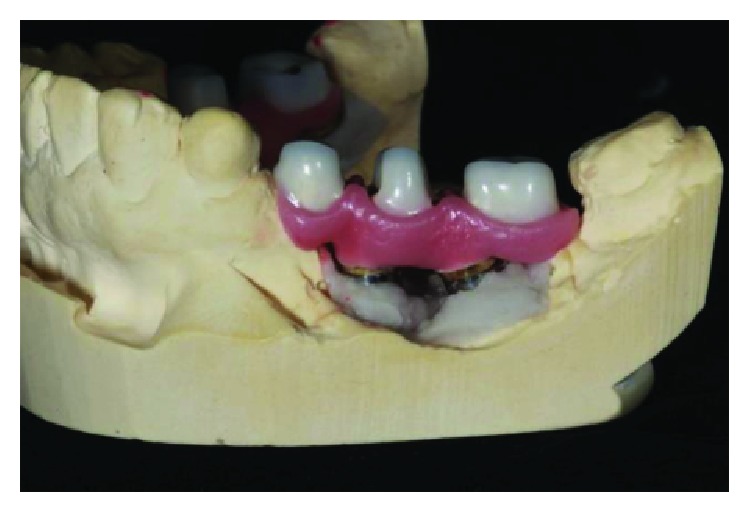
Application of feldspathic ceramic to the primary structure and application of a ceramic gingiva secondary structure, posterior left region. Source: Dental Design Laboratory.

**Figure 18:**
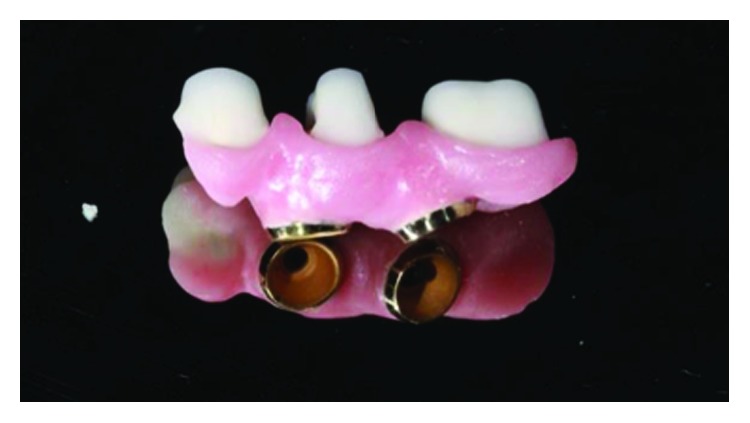
Gold bath in the connection-posterior left region. Source: own authorship.

**Figure 19:**
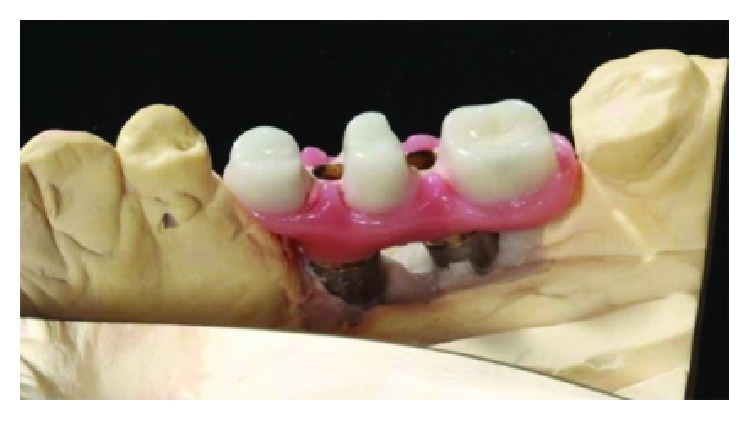
Space for insertion of the key. Source: own authorship.

**Figure 20:**
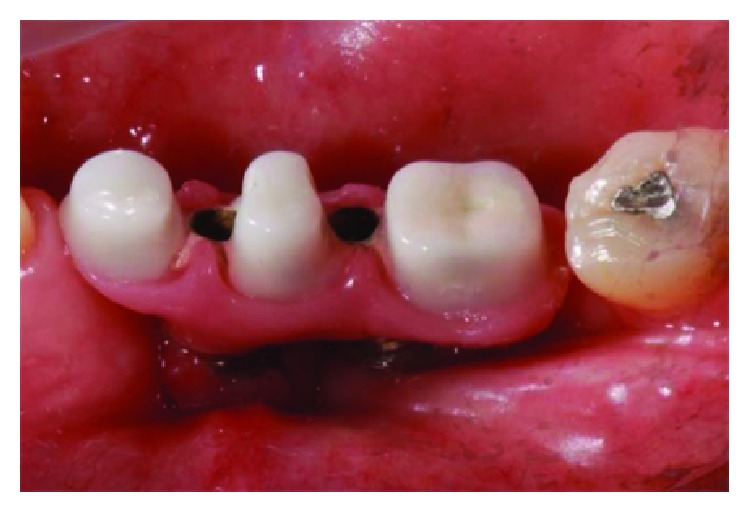
Primary and secondary structures in the mouth, posterior left region. Source: own authorship.

**Figure 21:**
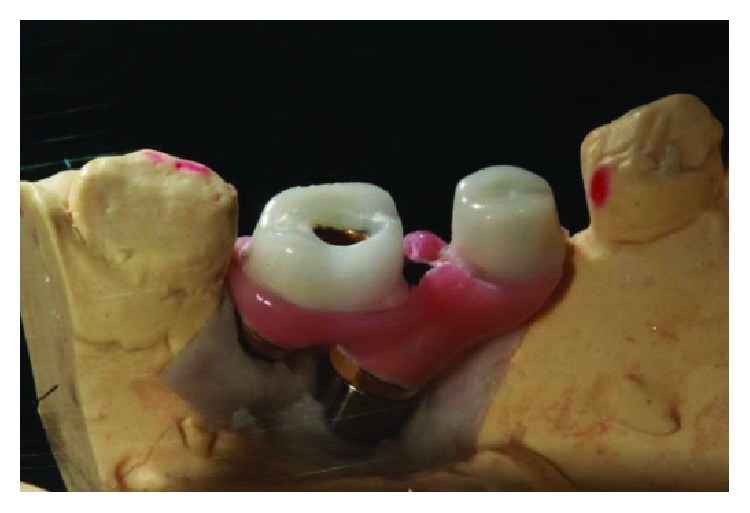
Application of feldspathic ceramic to the primary structure and application of a ceramic gingiva to the secondary structure, posterior right region. Source: own authorship.

**Figure 22:**
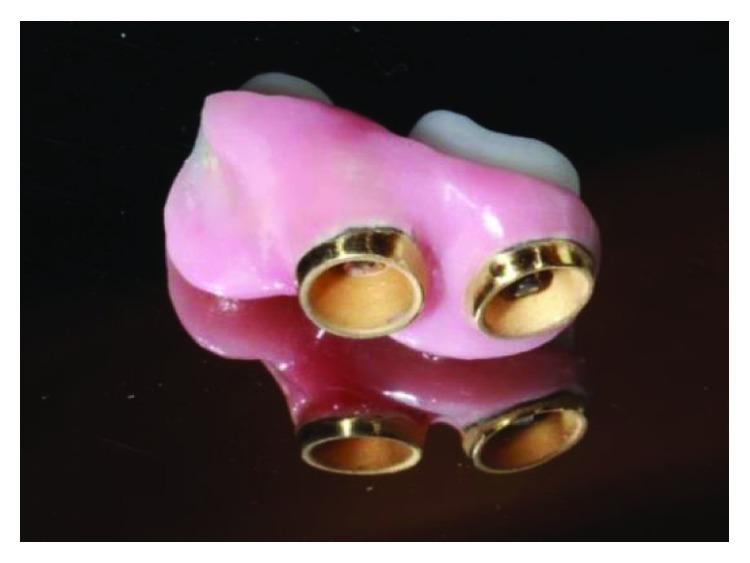
Gold bath in the connection-posterior right region. Source: own authorship.

**Figure 23:**
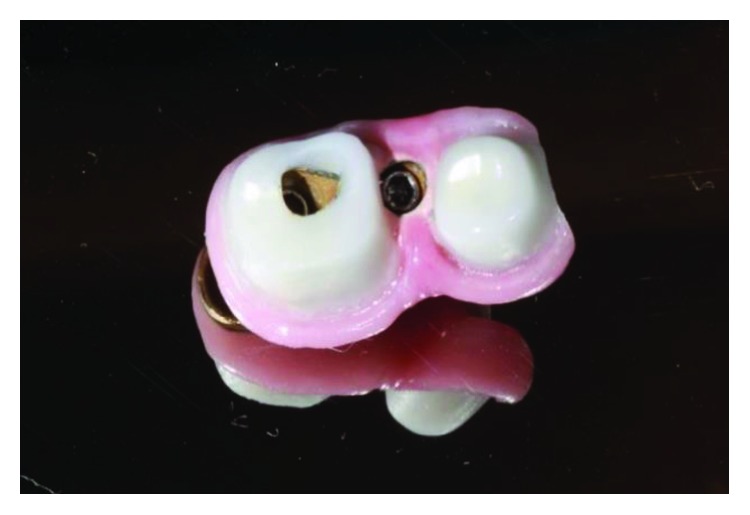
Space for insertion of the key, posterior right region. Source: own authorship.

**Figure 24:**
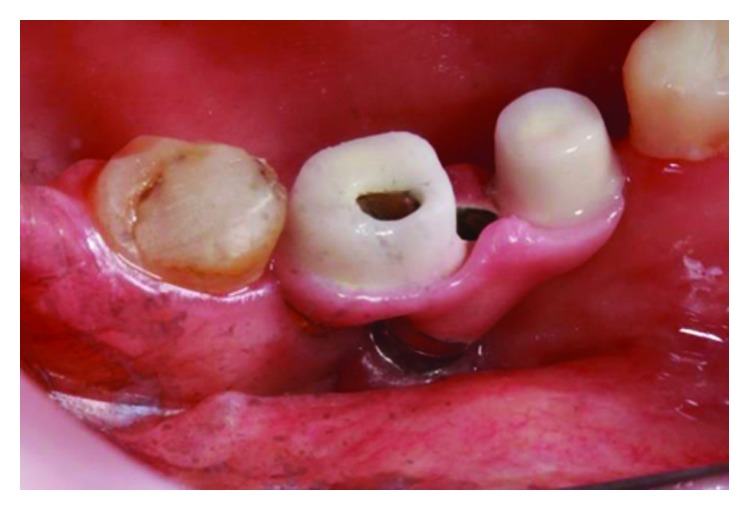
Primary and secondary structures in the mouth, posterior right region. Source: own authorship.

The aesthetic necessity of the final work resembled the rest of the lower arch, which was being rehabilitated with IPS e.max crowns (Ivoclar Vivadent), lithium disilicate, and the laboratory was asked to apply feldspathic ceramics to the metal structure in the region where the crowns would later be cemented. In the same way, the application of a ceramic gingiva—a secondary structure—was also necessary to restore any soft tissue lost (Figures 17 and 21). Figures 18 and 22 show the gold bath in the connection and Figures 19 and 23 the space for insertion of the key. The primary and secondary structure in the mouth can be seen in Figures 20 and 24.

**Figure 25:**
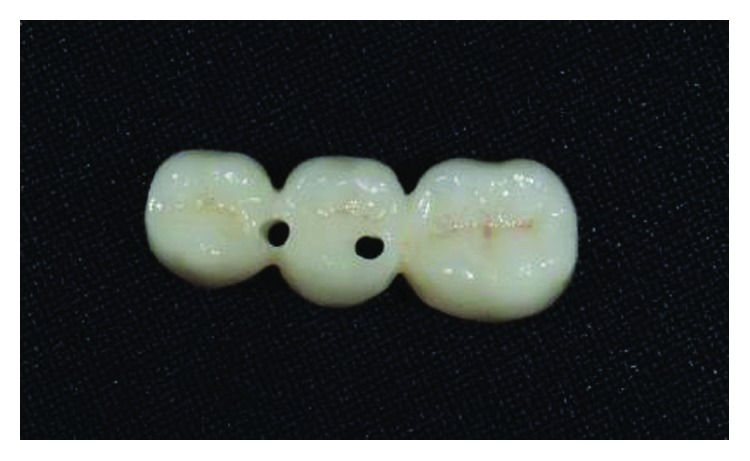
Finished crowns prior to installation in the mouth, posterior left region. Source: own authorship.

**Figure 26:**
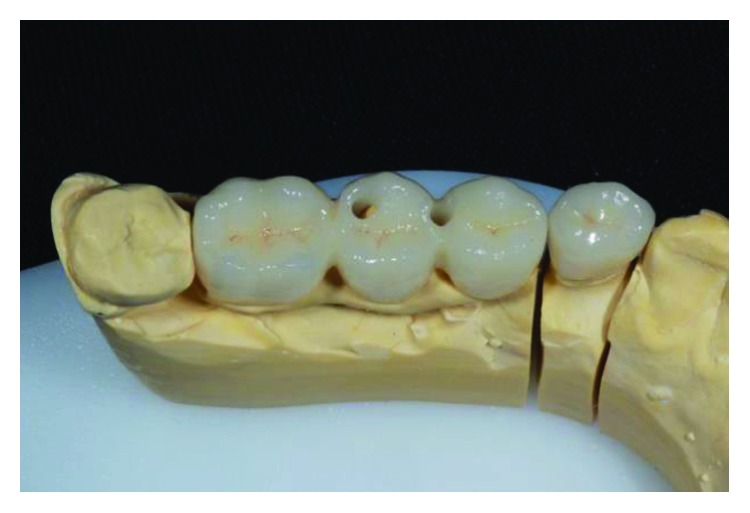
Finished crowns prior to installation in the mouth, posterior left region in plaster models. Source: own authorship.

**Figure 27:**
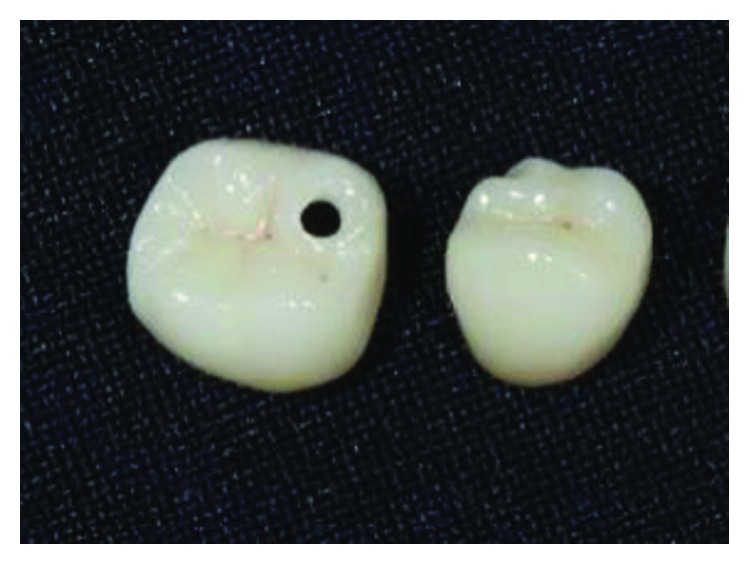
Finished crowns prior to installation in the mouth, posterior right region. Source: own authorship.

**Figure 28:**
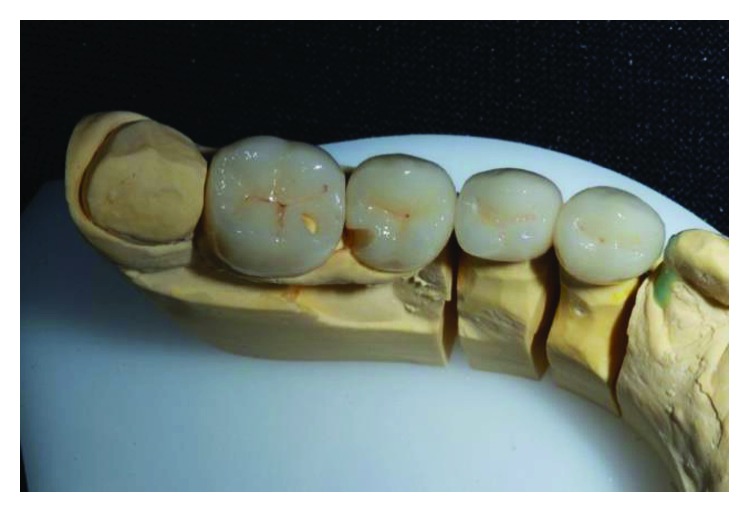
Finished crowns prior to installation in the mouth, posterior right region in plaster models. Source: own authorship.

The implant-supported ceramic crowns were confectioned in e.max lithium disilicate (Ivoclar Vivadent), and Figures 25–28 refer to finished crowns prior to installation in the mouth—in plaster models and outside them.

**Figure 29:**
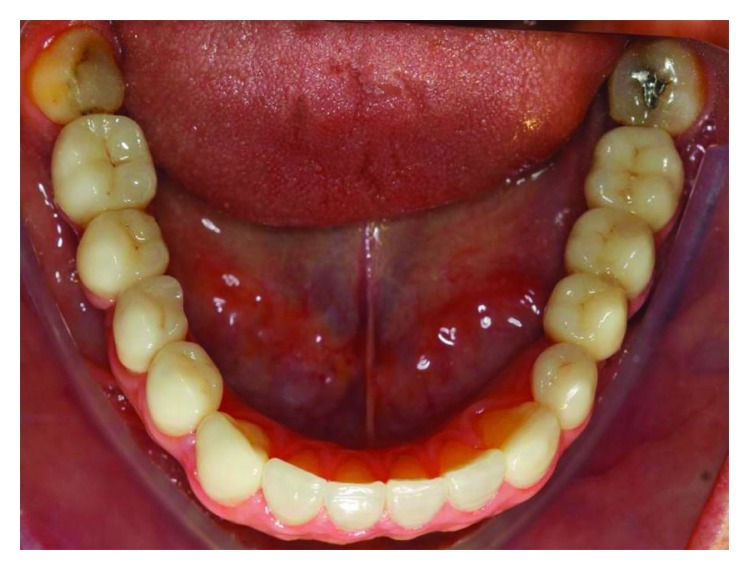
Occlusal view, final results. Source: DOCEO-SC.

**Figure 30:**
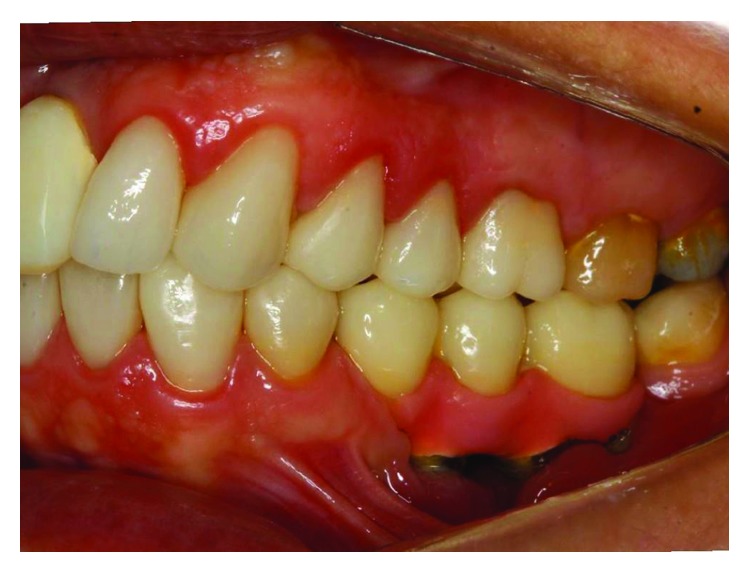
Lateral view, left posterior region, final results. Source: DOCEO-SC.

**Figure 31:**
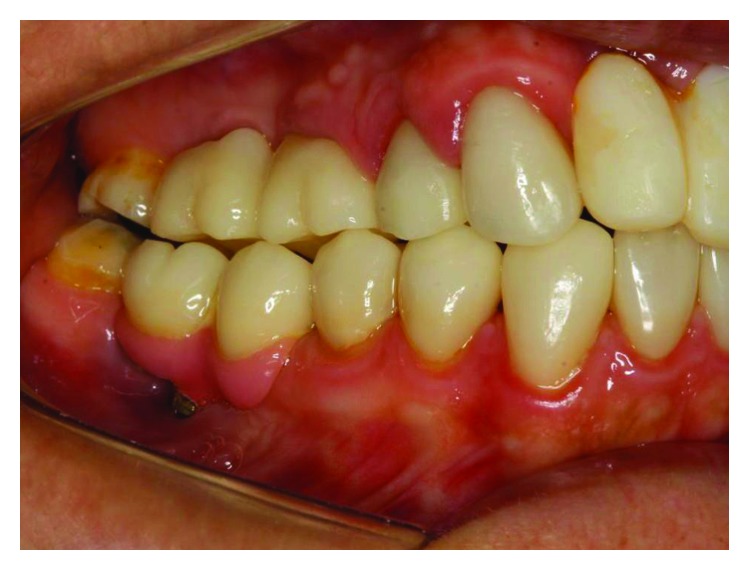
Lateral view, right posterior region, final results. Source: DOCEO-SC.

**Figure 32:**
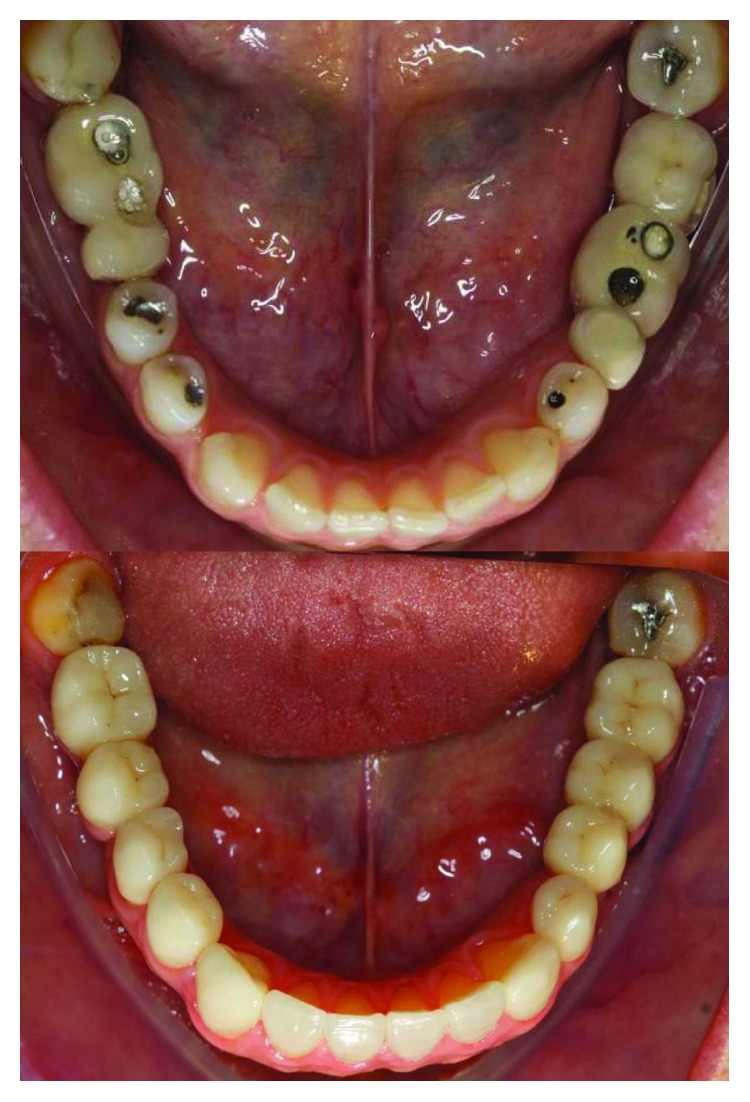
Comparison of the initial clinical case and the final results.

Fixation of crowns on the implant right side and left side followed the same protocol: the primary structure was screwed to a minipillar bolt with the manufacturer's established torque (foundry occurred in calcinable UCLAs with chromium-cobalt termination), and the secondary structure was cemented with Ultimate resin cement (3 M). During the cementation of the crowns, the occlusal orifices were protected with seal tape (polymer-polytetrafluoroethylene, patented by the commercial name Teflon, DuPont) so that the cement did not obstruct the existing space for a possible reintervention. After polymerization of the cement, the sealing tapes were maintained and the occlusal holes were restored with resin (3M Z-350). The final result can be seen in Figures 29–31 and achieved function and expected aesthetic.  Figure 32 shows the comparison of the initial clinical case and the final results.

## 3. Discussion

The clinical case described in the present article was customized to obtain function and aesthetics and mainly to solve a case of implants misaligned in the posterior region of the mandible since the patient did not agree to the removal of the already installed implants. The planning consisted of a combined cement-retained and screw-retained prosthesis system.

Peculiarities of each retention system should be considered and analyzed by the clinician at the time of choice. An important factor when a screwed prosthesis is chosen by the clinician is the retrievability [13, 14]. Retrievability is a factor directly related to the approach done in the present work which the screwed prostheses present. The possible need for reversal of a screwed prosthesis would be as follows: the need to maintain prosthetic components, loosening or fracture of the screws, abutment fracture, modification of the prosthesis after the loss of an implant, and surgical reinterventions [15]. However, it is important to be aware that repeated removal of the screwed prostheses may result in wear of the screw or implant, contributing to fracture of the component [16].

The installation of minipillars on the implants was necessary since implant sites with a band of ≤2 mm of keratinized mucosa showed to be more prone to brushing discomfort, plaque accumulation, and peri-implant soft tissue inflammation [17]. This fact occurs in select cases, particularly in the edentulous posterior mandible [18].

Another factor that we cannot fail to discuss is the question of passivity in the implant-supported prosthesis when we aim to maintain osseointegration and structural integrity [19]. These factors are fundamental, and many authors ensure that a cement-retained restoration is more likely to achieve passive fit than a screw-retained one. This increased passivity of cement-retained restorations rests on the assumption that the cement could act as a shock absorber and reduce stress to the bone and implant abutment structure [20]. The major cause of loss of prosthetic restorations on implants, bone loss of the ridge, fracture, or mobility of implants is nonpassive casting [21].The creation of a multiple-screwed prosthesis that has a passive seat in all the implants is difficult to achieve and, when this does not occur, will resort to an overload causing biomechanical and necrotic failures in the existing osseointegration [22].

Therefore, we can say that among all these factors addressed in the screwed prosthesis, the retrievability and the predictability of retention should be considered as main advantages, and in the cemented prosthesis, we can consider passivity as a main advantage [23, 24]. On this basis, our clinical case was planned like a reliable method to fabricate a retrievable cemented prosthesis.

In fact, it is understood that several factors influence the behavior of the prosthesis on implants, not only the retrievability but also retention and passivity. These include the diameter of the implants, length, surface design, and spatial positioning, among other factors. The types of connection systems between the implant and the prosthesis on the implant also vary and affect their performance, in addition to the question whether these prostheses are screwed or cemented [25].

In a recent review of the literature, the authors took an approach on the factors that would lead a clinician to decide on a cemented or screwed implant-supported prosthesis and identified three factors, which they called “determinants,” aesthetic outcome, retention, and biological risk. They found five factors which they called “conditioning factors”: passive fit, fracture strength, occlusal area, complications, and retrievability. They concluded that although there is no definite alternative for all clinical situations, the determining factors in certain cases may be decisive in the choice. For aesthetic reasons, when the implant angulation cannot be corrected to conceal the access hole, cementation is the system of choice. However, they make it clear that screwed implant-supported prosthesis is the best option when the interocclusal space is less than 6 mm or the implant margins cannot be located supragingivally or at the gingival level. The authors' orientation towards the best solution would be that in the absence of “determinant” factors, the decision should be based on “conditioning” factors, which vary depending on the type of prosthesis [26].

A recent research reports that the mode of retention of implanted crowns does not seem to affect their clinical correlates when considering parameters such as bleeding on probing, peri-implant bone loss, and inflammatory immune factor [27].

Combining both screw- and cement-retained restorations in the same prosthesis was introduced by using at least 1 screw retainer into a series of cement retainers within the same prosthesis [28, 29].

The purpose to create a natural similarity of a dental element to the prosthetic crown reproduced the optical properties of the dentin and enamel, justifying a customization of the pieces with respect to the application of feldspathic ceramics to the metallic structure [30, 31]. The use of an angled abutment was not used in the present clinical case due to the absence of suitable angled prosthetic components in the company catalog, thus accounting for burial of the implant in the region of element 37 and the use of a cantilever crown in this region.

In a 10-year retrospective analysis, a total of sixty cantilever prostheses were installed and monitored. During this period, there was no implant fracture, abutment, prosthesis, or porcelain. There was also no record of any soft tissue recession or radiographic evidence of bone loss. The results were positive since all sixty cantilevers installed remained satisfactory [32].

The combination of a cement-retained and screw-retained prosthesis system was planned and developed to address the need for the clinical case in question.

## 4. Conclusion

In evaluating the literature on cemented and screwed implant-supported prostheses, we understand that the two options have similar survival rates and complications, with comparable bone and soft tissue levels. The screw-retained crowns have a predictable retention and are reversible for possible maintenance, in addition to presenting no problems associated with excess cement. Already, implants with inadequate angulation, therefore, continue to be a relevant indication for cement-retained prostheses, aiming for passivity, aesthetics, and retrievability. The combination of screwed and cemented options for malpositioned implants in the mandible posterior region would undoubtedly be a good option, taking into account the aesthetic and functional parameters as long as greater scientific evidence was described in the literature.
